# Alginate-Derived Oligosaccharide Inhibits Neuroinflammation and Promotes Microglial Phagocytosis of β-Amyloid

**DOI:** 10.3390/md13095828

**Published:** 2015-09-16

**Authors:** Rui Zhou, Xu-Yang Shi, De-Cheng Bi, Wei-Shan Fang, Gao-Bin Wei, Xu Xu

**Affiliations:** 1 Shenzhen Key Laboratory of Marine Bioresources and Ecology, Collage of Life Science, Shenzhen University, Shenzhen 518060, China; E-Mails: zhouruiswg@gmail.com (R.Z.); bidecheng@foxmail.com (D.-C.B.); 2 College of Life Science, Shenzhen Key Laboratory of Microbial Genetic Engineering, Shenzhen University, Shenzhen 518060, China; E-Mails: shixuyang075@gmail.com (X.-Y.S.); fangweishan90@163.com (W.-S.F.); weigaobin@126.com (G.-B.W.)

**Keywords:** alginate, β-amyloid, microglia, neuroinflammation, phagocytosis, toll-like receptor 4

## Abstract

Alginate from marine brown algae has been widely applied in biotechnology. In this work, the effects of alginate-derived oligosaccharide (AdO) on lipopolysaccharide (LPS)/β-amyloid (Aβ)-induced neuroinflammation and microglial phagocytosis of Aβ were studied. We found that pretreatment of BV2 microglia with AdO prior to LPS/Aβ stimulation led to a significant inhibition of production of nitric oxide (NO) and prostaglandin E_2_ (PGE_2_), expression of inducible nitric oxide synthase (iNOS) and cyclooxygenase-2 (COX-2) and secretion of proinflammatory cytokines. We further demonstrated that AdO remarkably attenuated the LPS-activated overexpression of toll-like receptor 4 (TLR4) and nuclear factor (NF)-κB in BV2 cells. In addition to the impressive inhibitory effect on neuroinflammation, we also found that AdO promoted the phagocytosis of Aβ through its interaction with TLR4 in microglia. Our results suggested that AdO exerted the inhibitory effect on neuroinflammation and the promotion effect on microglial phagocytosis, indicating its potential as a nutraceutical or therapeutic agent for neurodegenerative diseases, particularly Alzheimer’s disease (AD).

## 1. Introduction

In the last decade, increasing evidence has demonstrated that neuroinflammation is involved in the pathogenesis and progression of various neurodegenerative disorders, including Alzheimer’s disease (AD) [1] and Parkinson's disease [2]. The accumulation of β-amyloid (Aβ) and the accompanying neurotoxicity is considered to be one of the most important pathologies of AD [3]. Microglial cells are resident immune cells and act as effectors of various processes in normal and pathological brains. Two of the main functions of microglial cells are mediating neuroinflammation and clearing toxic Aβ aggregates via phagocytosis [4]. Previous reports have proposed that activated microglial cells are induced by pathogen-associated environmental toxins, such as lipopolysaccharide (LPS) or Aβ [5]. LPS/Aβ activates microglial cells via various receptors expressed on the cell surfaces, especially toll-like receptors (TLR) [6]. TLR activation of the microglial cells induce the secretion of a number of inflammatory factors that lead to cytotoxic effects and brain damage, resulting in serious neuroinflammation [7,8]. The TLR4 signaling that triggers the generation of the inflammatory mediators depends on the activation of multiple intracellular signaling pathways, including nuclear factor (NF)-κB and mitogen-activated protein kinases (MAPKs) [6,9]. It has been established that agents that can efficiently attenuate TLR signaling and decrease the inflammatory responses of the activated microglial cells are beneficial in treating AD [10]. In addition, microglia are essentially the macrophages of the brain, and phagocytosis is a key feature of these cells. This function supports brain homoeostasis by clearing neurotoxic substances, including cellular debris and Aβ aggregates. However, it has been found that the clearance of toxic Aβ aggregates by the microglia is impaired in AD [11]. Considering the inflammatory and phagocytotic functions of microglia, one possible therapeutic method for treating AD is to reduce microglia-mediated neuroinflammation and increase microglial clearance of Aβ [12].

**Figure 1:**
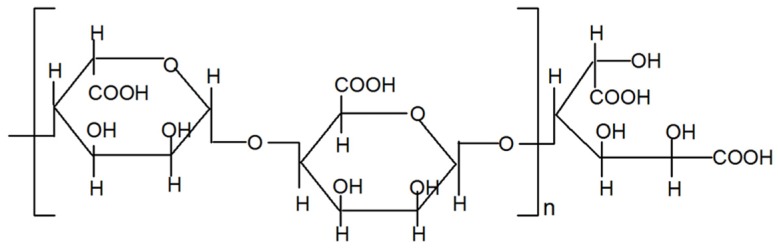
Schematic representation of chemical structures of alginate-derived oligosaccharide (AdO) prepared by oxidative degradation.

Alginate derived from various marine brown algae is an acidic polysaccharide composed of alternating blocks of β-(1-4)-d-mannuronic acid (M) and α-(1-4)-l-guluronic acid (G). Alginate has been widely applied in biotechnology for microencapsulation, drug delivery, and tissue engineering [13,14]. Alginate-derived oligosaccharide (AdO) produced by depolymerizing the polysaccharide using various degradation methods shows a variety of biological activities. Unsaturated AdO depolymerized by enzymatic depolymerization exerts anti-tumor [15], anti-oxidant [16,17], and immunomodulatory effects [18,19], whereas the saturated AdO prepared by acid hydrolysis posses low bioactivities. The anti-inflammatory activities of alginate have been described [20,21]. AdO prepared by oxidative degradation possibly has a carboxyl group at the 1-position of the reducing end, which have been characterized in the previous work (Figure 1) [22]. We have demonstrated that AdO prepared by oxidative degradation suppressed the inflammatory response in LPS-activated marine macrophage RAW 264.7 cells [23], which inspired us to investigate whether AdO could reduce neuroinflammation and subsequently attenuate neuroinflammation-mediated diseases.

In the current study, our aim is to investigate the molecular mechanisms of the inhibitory effect of AdO on LPS/Aβ-stimulated microglial neuroinflammation, and to demonstrate the effect of AdO on the phagocytosis of Aβ by BV2 microglia. Our findings suggested the potential application of AdO as a therapeutic agent for the treatment of AD.

## 2. Results

### 2.1. AdO Suppresses LPS-Induced Production of Inflammatory Mediators in BV2 Cells

**Figure 2:**
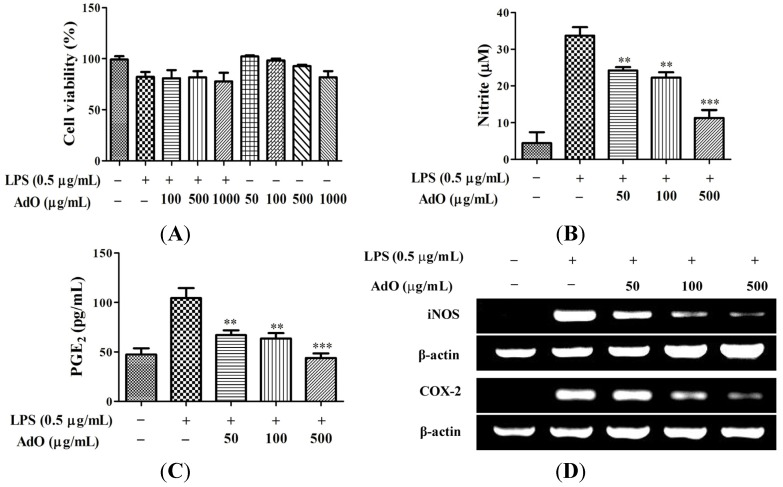

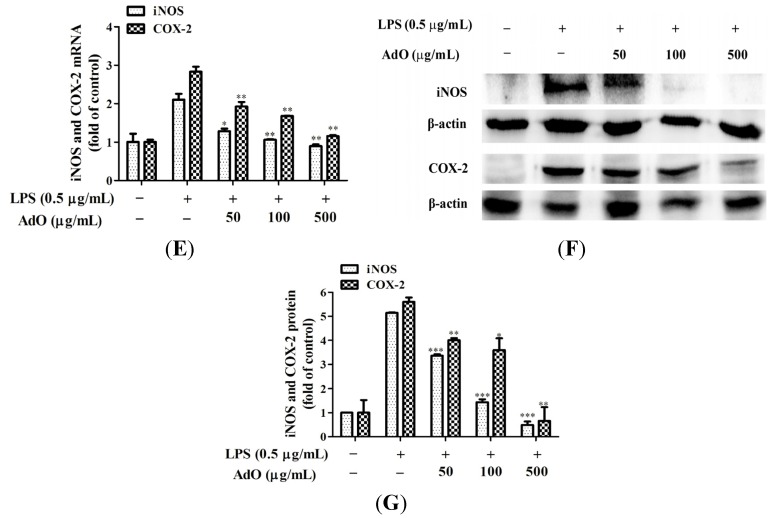
AdO reduced the lipopolysaccharide (LPS)-activated production of nitric oxide (NO) and prostaglandin E_2_ (PGE_2_) and the expression of inducible nitric oxide synthase (iNOS) and cyclooxygenase-2 (COX-2) in BV2 microglial cells. (**A**) Cell viability was evaluated using the CCK-8 assay for control cells, LPS (0.5 μg/mL) treatment cells, LPS with AdO (100–1000 μg/mL) treatment cells, and AdO (50–1000 μg/mL) treatment cells; (**B**) The nitrite concentration was measured as an indicator of NO production using the Griess reagent; (**C**) PGE_2_ production was analyzed using ELISA; (**D**) The expression of the iNOS and COX-2 mRNAs was detected by RT-PCR; (**E**) The relative mRNA levels of iNOS and COX-2 were analyzed with reference to the control group; (**F**) The expression of the iNOS and COX-2 proteins was detected using Western blot analysis; (**G**) The relative levels of the iNOS and COX-2 proteins were analyzed with reference to the control group. The data are presented as the mean ± SD for three independent experiments. * *p* < 0.05, ** *p* < 0.01 and *** *p* < 0.001 indicate significant differences compared with the LPS-treated group.

To evaluate the toxicity of AdO to BV2 cells, the cell viability was measured after treatment with AdO. Results showed that AdO with concentrations lower than 500 μg/mL led to little cytotoxic effect in BV2 cells (Figure 2A). Therefore, we investigated the anti-neuroinflammatory effect of 50–500 μg/mL of AdO in LPS-activated BV2 cells in this study. Next, we evaluated the effect of AdO on the production of nitric oxide (NO) and prostaglandin E_2_ (PGE_2_) by LPS-activated BV2 cells. As shown in Figure 2B and C, AdO at various concentrations inhibited the LPS-induced NO and PGE_2_ levels in BV2 cells in a dose-dependent manner. On the basis that inducible nitric oxide synthase (iNOS) and cyclooxygenase-2 (COX-2) are the key enzymes for production of NO and PGE_2_, respectively, we performed RT-PCR and Western blot analyses to determine the effects of AdO on the expression of iNOS and COX-2. The results showed that the LPS-induced expression of iNOS and COX-2 was suppressed by AdO treatment at the mRNA level (Figure 2D, E) and at the protein level (Figure 2F,G). These results indicated that AdO reduces NO and PGE_2_ production by inhibiting the iNOS and COX-2 expression.

### 2.2. AdO Inhibits LPS/Aβ-Activated Secretion of Inflammatory Cytokines in BV2 Cells

**Figure 3:**
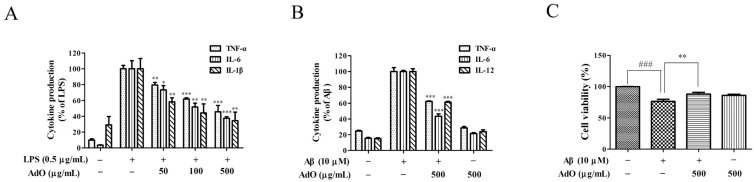
AdO inhibited the LPS/Aβ-activated secretion of proinflammatory cytokines. (**A**) Tumor necrosis factor-α (TNF-α), interleukin (IL)-1β and IL-6 expression in the LPS-activated BV2 cells was measured by ELISA; (**B**) TNF-α, IL-6 and IL-12 production in the Aβ-activated BV2 cells was evaluated by ELISA; (**C**) The cell viability following AdO (500 μg/mL) pretreatment with or without Aβ (10 μM) stimulation was detected by the CCK-8 assay. The data are presented as the mean ± SD for three independent experiments. * *p* < 0.05, ** *p* < 0.01 and *** *p* < 0.001 indicate significant differences compared with the LPS/Aβ-treated group, and ### *p* < 0.001 indicates a significant difference between the control group and the Aβ-treated group.

As demonstrated in Figure 3, the secretion of tumor necrosis factor-α (TNF-α), interleukin (IL)-1β and IL-6 (Figure 3A) was notably increased with LPS stimulation, but considerably decreased in a dose-dependent manner in the AdO-pretreated cells. Next, we investigated whether AdO could affect the Aβ-activated production of the inflammatory cytokines by the BV2 cells. As expected, the results showed that the AdO treatment remarkably reduced the Aβ-stimulated production of TNF-α, IL-6 and IL-12 (Figure 3B). Interestingly, we demonstrated that Aβ reduced the cell viability to 76.6% ± 3.36%, but pre-treatment with AdO exerted a positive effect on the cell viability (Figure 3C). These results suggest that AdO could effectively suppress the LPS/Aβ-activated inflammatory cytokines secretion by the BV2 cells.

### 2.3. AdO Inhibits LPS-Activated Signaling Pathway in BV2 Cells

**Figure 4:**
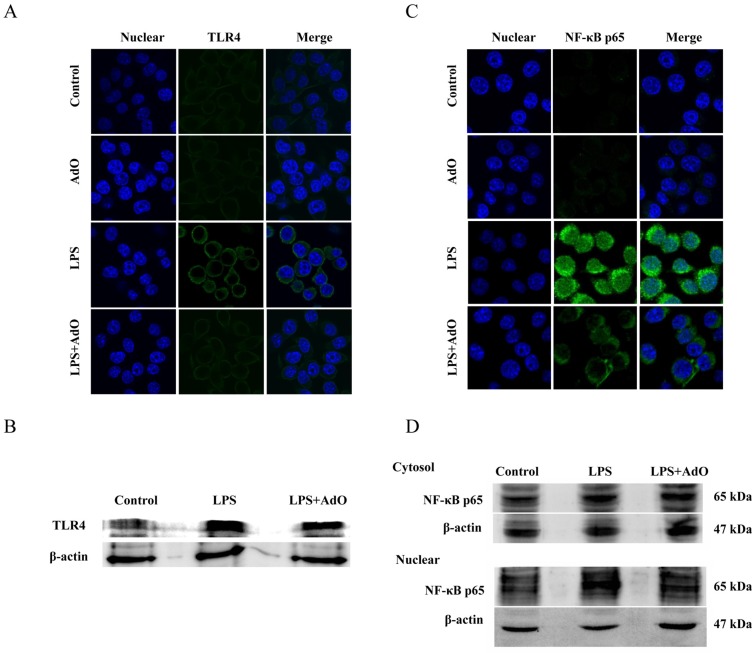
AdO suppressed the LPS-induced toll-like receptor 4 (TLR4) expression and nuclear factor (NF)-κB activation. TLR4 expression was evaluated using immunofluorescence analysis (**A**) and Western blot analysis (**B**); (**C**) NF-κB p65 expression was examined using immunofluorescence analysis; (**D**) The cytoplasm and nuclear proteins were extracted and the NF-κB p65 protein was analyzed by Western blot analysis. The immunofluorescence analysis was carried out by laser scanning confocal microscopy (60×), and the images were processed using ImageJ software. The immunofluorescence analysis and Western blot analysis were performed in three independent experiments.

Next, we evaluated the effect of AdO on the LPS-activated TLR4-NF-κB signaling pathway using immunofluorescence and Western blot analysis. As shown in Figure 4A, TLR4 expression was notably increased by LPS stimulation, and this effect was blocked by the addition of AdO. Similar results were observed in the Western blot analysis (Figure 4B). Figure 4C clearly shows that the translocation of the NF-κB/p65 subunit from the cytoplasm to the cell nucleus was augmented by LPS-stimulation and reduced by AdO treatment. The results of the Western blot analysis agreed well with the immunofluorescence analysis (Figure 4D).

### 2.4. Effect of AdO on LPS/Aβ-Activated Morphological Changes of the BV2 Cells

**Figure 5:**
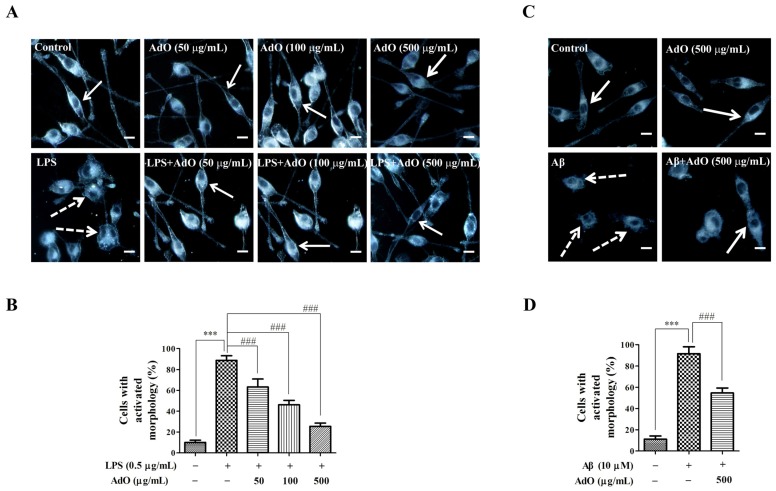
AdO inhibited the morphological changes induced by LPS/β-amyloid (Aβ) in the BV2 cells. (**A**) The cellular morphology of the control group, AdO (50–500 μg/mL)-treated group, LPS (0.5 μg/mL)-treated group and the group treated with AdO prior to LPS are shown in the dark field images; (**B**) The percentage of cells exhibiting the activated morphology was statistically analyzed; (**C**) The cellular morphology of the control group, AdO (500 μg/mL)-treated group, and Aβ (10 μM)-treated group as well as the group treated with AdO prior to Aβ are shown in the dark field images; (**D**) The percentage of cells exhibiting the activated morphology was statistically analyzed. The cellular morphology was observed using dark-field microscopy (40×), and the images were analyzed using ImageJ software (National Institutes of Health, Bethesda, MD, USA). The normal cell morphology is indicated by white arrows, and the activated cell morphology is indicated by white dotted arrows. Scale bar = 20 μm. The images were from three independent experiments. *** *p* < 0.001 indicates significant differences between the control group and the LPS/Aβ-treated group; ### *p* < 0.001 indicates significant differences between the LPS/Aβ-treated group and the group treated with AdO prior to LPS/Aβ.

Furthermore, it has been demonstrated that anti-inflammatory agents can protect the microglia from LPS or Aβ-activated morphological changes [24,25]. Here, the effect of AdO on the LPS/Aβ-activated cell morphological changes was observed using dark-field microscopy. The enlargement of microglial cell bodies and an amoeboid morphology with retraction of extensions are generally induced by LPS [24]. While these changes were obvious in the LPS-activated BV2 microglia (see white dot arrows in Figure 5A), AdO markedly suppressed those morphological changes (Figure 5A). We analyzed the number of cells with normal (white arrows) or activated (white dot arrows) morphology in ten randomly selected images. The results showed that pretreatment with AdO prevented the LPS-activated morphological changes of the cells in a dose-dependent manner (Figure 5B). In addition, the effect of AdO on the Aβ-activated microglia morphological changes was investigated. As expected, AdO protected the microglia from the Aβ-activated morphological changes (Figure 5C). The analyzed results are shown as histograms (Figure 5D).

### 2.5. AdO Promotes the Phagocytosis of Aβ in BV2 Cells

**Figure 6:**
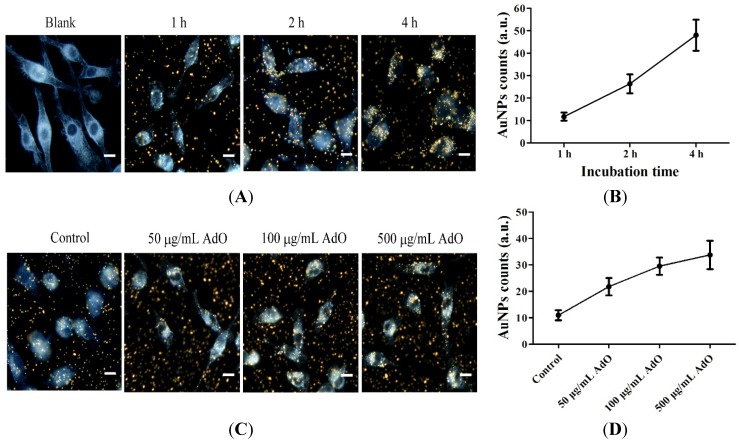

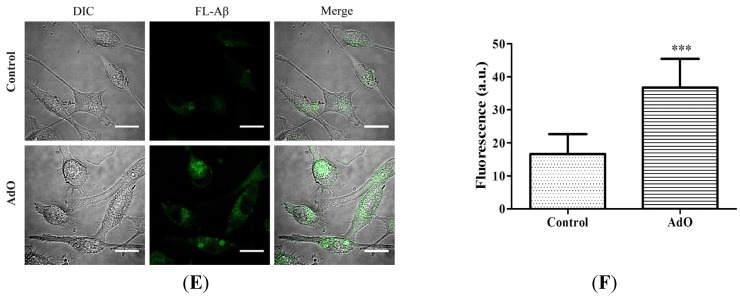
AdO promoted the phagocytosis of gold nanoparticles (AuNPs) and Hilyte Fluo™ 488-labled β-amyloid (1-42) (FL-Aβ) in the BV2 cells. (**A**) The cells were treated with 1 pM of the AuNPs for indicated incubation times; (**B**) The phagocytosis of the AuNPs by the BV2 cells was evaluated by counting the number of AuNPs in fifty cells using ImageJ software; (**C**) The cells were treated with AdO (50–500 μg/mL) for 20 h and then incubated with 1 pM of the AuNPs for 1 h. (**D**) The average number of phagocytosed AuNPs in fifty cells was analyzed using ImageJ software. The cells with the accumulated AuNPs were examined using dark-field microscopy (40×), scale bar = 20 μm; (**E**) The cells were treated with AdO (50 μg/mL) for 20 h and then incubated with 500 nM FL-Aβ for 4 h. The cells with the accumulated FL-Aβ were examined using laser scanning confocal microscopy (60×), and the morphology of the cells was shown using differential interference contrast (DIC) images, scale bar = 20 μm; (**F**) The average fluorescent intensity of fifty cells was evaluated using ImageJ software. The microscopic images were from three independent experiments. *** *p* < 0.001 indicates significant differences compared with the control group.

First, gold nanoparticles (AuNPs) with a 100-nm diameter were used to evaluate the non-specific phagocytic ability of BV2 cells. The BV2 microglia were incubated with AuNPs for the indicated times and observed using dark-field microscopy. The cells with phagocytosed AuNPs are shown in Figure 6A. The intracellular AuNPs were analyzed using ImageJ software. The results showed that BV2 microglia readily accumulated increasing amounts of AuNPs in a time-dependent manner (Figure 6B). Next, the BV2 cells were treated with AdO for 20 h, after which AuNPs were added, and the incubation was continued for an additional 1 h. We found that the BV2 cells treated with AdO accumulated more AuNPs than the untreated cells (Figure 6C). The analyzed results are shown in Figure 6D. AdO treatment increased the phagocytosis of AuNPs in a concentration-dependent manner, suggesting that AdO activated the BV2 microglia and promoted phagocytosis. In addition, the results clearly showed that AdO promoted the uptake of AuNPs in a concentration-dependent manner at the single cell level (Supplementary information Figure S1). These results demonstrated that AdO could promote non-specific phagocytosis in microglia. Next, Hilyte Fluo™ 488-labled β-amyloid (1-42) (FL-Aβ) was used to evaluate the specific phagocytic ability of BV2 cells. We utilized fluorescence analysis to determine the effect of AdO on the microglial phagocytosis of FL-Aβ. As shown in Figure 6E, the control cells engulfed a small amount of FL-Aβ, whereas the AdO-treated cells accumulated a larger amount of FL-Aβ. Compared to the fluorescence (16.6 ± 5.9) of the untreated cells, the AdO treatment of the cells promoted the uptake of FL-Aβ up to two-fold (36.7 ± 8.6) (Figure 6F).

### 2.6. TLR4 Is Involved in the AdO-Promoted Microglial Phagocytosis

**Figure 7:**
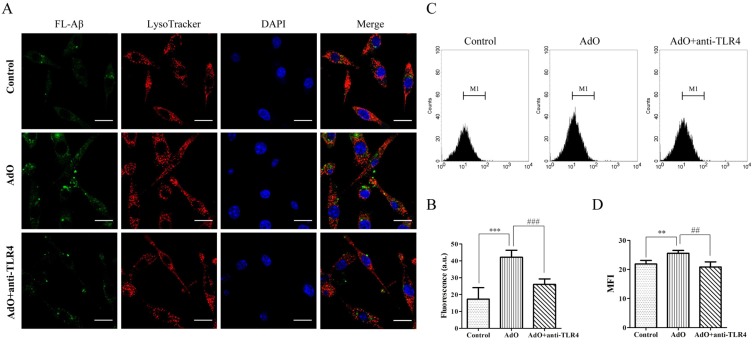
The involvement of TLR4 in the AdO-promoted phagocytosis of Aβ in the BV2 cells. (**A**) The cells were treated with anti-TLR4 (10 μg/mL) for 1 h prior to AdO treatment for 20 h and then incubated with 500 nM FL-Aβ for 4 h. The lysosomes and nuclei of the cells were stained with LysoTracker Red DND-99 and 4′,6-diamidino-2-phenylindole (DAPI), respectively. The cells with the accumulated FL-Aβ were observed using laser scanning confocal microscopy (60×), scale bar = 20 μm; (**B**) The average fluorescence intensity of fifty cells in each group was evaluated using ImageJ software; (**C**) The phagocytosis of FL-Aβ in untreated cells, AdO-treated cells, and anti-TLR4 and AdO-treated cells was analyzed using flow cytometry. The flow cytometric plots show the M1 region of the phagocytic cell populations based on fluorescence intensity; (**D**) The mean fluorescence intensity (MFI) of the cells in the M1 region is shown. The images were from three independent experiments. ** *p* < 0.01 and *** *p* < 0.001 indicate significant differences between the control group and the AdO-treated group, and ## *p* < 0.01 and ### *p* < 0.001 indicate significant differences between the AdO-treated group and the AdO combined with anti-TLR4-treated group.

TLR4 is the most important receptor for uptake and clearance of Aβ in the BV2 cells [26]. To determine whether TLR4 is involved in the AdO-augmented microglial phagocytosis of Aβ, we used a TLR4 antibody to block TLR4 as mentioned in the previous reports [27,28]. The results indicated that the addition of the anti-TLR4 antibody suppressed the AdO-induced phagocytosis of FL-Aβ (Figure 7A): The incorporation was significantly less than that of the cells treated with AdO alone (Figure 7B). These results indicated that TLR4 is involved in AdO-promoted microglial phagocytosis. In the merged images (Figure 7A), the distribution of FL-Aβ was observed in the cytoplasm and lysosomes, suggesting that the FL-Aβ had already entered the cells. Furthermore, we conducted a flow cytometry assay to verify this involvement. The flow cytometric plots of the phagocytic cell populations are shown in Figure 7C, and the mean fluorescent intensity (MFI) showed a trend that was consistent with the fluorescence microscopic analysis (Figure 7D). Our flow cytometric assay results further confirmed that TLR4 is involved in the AdO-induced promotion of microglial phagocytosis. Finally, because the lysosomes and cytoskeleton participate in the phagocytotic process [29], we evaluated the effect of AdO on the lysosomes and cytoskeleton of the BV2 cells. Unfortunately, these results demonstrated that AdO had little effect on the lysosome production and cytoskeleton formation compared with the untreated cells (Figure S2).

## 3. Discussion

Agents that either inhibit the microglia-mediated neuroinflammation or enhance the microglial phagocytosis would be beneficial to AD therapy [5,12,30]. Numerous studies have demonstrated that natural products play important roles in treating or slowing the progression of neurodegenerative disease [30,31,32]. However, several natural products were found to exert neuroprotection by simultaneously suppressing microglial activation and promoting microglial phagocytosis. Blueberries have been found to significantly attenuate microglial activation and enhance microglial clearance of Aβ [33]. Curcumin exerts excellent neuroprotection through its anti-inflammatory activity and its promotion of phagocytosis [27]. Therefore, it is significant to find more natural products that appropriately inhibit microglial activation and promote the microglial uptake of Aβ. Polysaccharides from natural products exert notable neuroprotective and anti-neuroinflammatory activity [32,34]. Alginate is a natural polysaccharide that is found in various marine brown seaweeds. There have been significant studies in the last few decades that have revealed the bioactivities and broadened the utility of alginate-derived oligosaccharide [13,14]. Previous work has demonstrated that alginate oligosaccharides (1300 Da) prepared by enzymatic depolymerization could easily cross the blood–brain barrier (BBB) [35,36]. We have shown that alginate oligosaccharides prepared by enzymatic and oxidative degradation exhibited similar degree of polymerization (DP) (representative of its molecular mass) using thin-layer chromatography (TLC) analysis in our previous work [18]. The average molecular weight of AdO is about 1500 Da. These findings indicated the accessibility of AdO to the BBB and suggested the potential application of AdO for treating neurodegenerative diseases. In our previous study, we found that AdO could remarkably reduce the production of inflammatory mediators in LPS-activated macrophages [23], which encouraged us to study its anti-neuroinflammatory activity. In the present work, we aimed to evaluate the effects of AdO on the neuroinflammatory responses and microglial phagocytosis of Aβ.

The production of NO and PGE_2_ and the expression of iNOS and COX-2 are the most important processes involved in LPS-activated neuroinflammation [34]. Here, we found that AdO pretreatment effectively inhibited the LPS-activated production of NO and PGE_2_ via the suppression of the transcriptional activation of iNOS and COX-2 in BV2 cells (Figure 2). Excessive production of pro-inflammatory cytokines is considered to be an initiator of neuroinflammatory responses, which is a hallmark of neurodegenerative disease [37], and to cause neuronal cytotoxicity and induce nerve cell damage. This study proved that AdO-treatment of BV2 cells remarkably inhibited the LPS-activated production of TNF-α, IL-6 and IL-1β as well as the Aβ-activated production of TNF-α, IL-6 and IL-12 (Figure 3). These results suggest that AdO may be useful in the treatment of neuroinflammation by suppressing microglial activation and attenuating the production of inflammatory mediators.

It has been demonstrated that TLR4-mediated activation of NF-κB signaling pathway plays an important role in the neuroinflammatory responses and various neurodegenerative diseases [9]. The current work showed that pretreatment of BV2 cells with AdO could remarkably suppress the LPS-stimulated TLR4 expression, indicating that the TLR4 signaling pathway was involved in the anti-neuroinflammatory effect of AdO (Figure 4). NF-κB is kept inactive through the binding of IκB proteins in resting microglial cells. Microglial activation with LPS activates NF-κB signaling, leading to IκB phosphorylation and degradation, and the subsequent NF-κB subunit nuclear translocation [38]. Here, we found that the nuclear translocation of the NF-κB p65 subunit was effectively attenuated by AdO treatment (Figure 4). These findings indicate that the inhibitory effects of AdO on TLR4 expression may involve the inactivation of NF-κB signaling in LPS-activated BV2 microglia, subsequently leading to the suppression of inflammatory mediator production. In addition, we found that AdO could protect the microglia from the LPS/Aβ-activated morphological changes (Figure 5). In either case, these results indicated that the neuroprotective effect of AdO is mediated by inactivation the TLR4-NF-κB signaling pathway.

Another key role of microglial cells is their capability to clear toxic Aβ aggregates. Aβ accumulation plays an important role in the progression of AD [3]. Under normal physiological conditions, Aβ aggregates are adequately cleared and degraded by the microglia and macrophages in the brain. Unfortunately, impaired phagocytosis of Aβ by the microglia and macrophages is observed in AD and is considered to be one of the pathological hallmarks of this disease [39]. After demonstrating the excellent inhibitory effect of AdO on microglia-mediated neuroinflammation, we further explored the effect of AdO on the phagocytosis by BV2 cells. Interestingly, we found that AdO treatment promoted the phagocytosis of FL-Aβ (Figure 6). Because TLR4 is directly or indirectly activated to induce the uptake of toxic Aβ aggregates [26,40], we evaluated the involvement of TLR4 in the promotional effect of AdO on the microglial uptake of Aβ. Blocking TLR4 with anti-TLR4 prior to AdO treatment resulted in a decrease in the phagocytosis of Aβ, indicating that AdO promoted phagocytosis through an interaction with TLR4 (Figure 7).

Neuroinflammation and microglial phagocytosis are both important factors to study with respect to the therapeutic intervention for AD [4]. Therefore, the development of natural products that cannot only inhibit the neuroinflammation but that can also enhance the clearance of Aβ is important for the prevention and treatment of AD. It was interesting to find that AdO exhibited remarkably inhibitory effect on neuroinflammation and promoted microglial phagocytosis of Aβ in this study. We demonstrated that AdO inhibited the neuroinflammatory response of LPS-activated BV2 cells, possibly by attenuating TLR4 expression and inactivating the NF-κB signaling pathway. However, the molecular mechanism by which AdO promoted the phagocytosis of Aβ is still uncertain. Our results indicated that the AdO-induced promotion of the uptake of Aβ may directly or indirectly involve TLR4. It has been demonstrated that TLR4 participates in the neuroinflammatory process, suggesting that TLR4 activation aggravates neuroinflammation-mediated diseases [41]. In contrast, studies with mice expressing a mutated form of TLR4 suggested that the activation of microglial TLR4 could reduce Aβ accumulation [42], indicating that TLR4 activation produced the neuroprotective effect. Whether TLR4 activation is neurotoxic or neuroprotective may differ among the various pathological conditions. The present results support the concept that TLR4 activation is tightly controlled to regulate its different roles in neuroinflammation and microglial phagocytosis. Further studies will focus on the molecular mechanisms of the dual effect of AdO on microglial cells in depth and explore the therapeutic potential of AdO in an in vivo model of AD.

## 4. Materials and Methods

### 4.1. Materials

Sodium alginate (15-20 cps grade), FITC-phalloidin and 4′,6-diamidino-2-phenylindole (DAPI) were purchased from Sigma-Aldrich (St. Louis, MO, USA). H_2_O_2_ was supplied by Chengdu Kelong Chemical Co., Ltd. (Chengdu, China). Fetal bovine serum (FBS) was obtained from Biontex (Planegg, Germany). Dulbeco’s Modified Eagle’s Medium (DMEM) was purchased from Thermo Scientific (Hudson, NH, USA). A CCK-8 kit was supplied from Beyotime Inst Biotech (Jiangsu, China). Aβ oligomers were prepared using amyloid-β 1-42 peptide (ChinaPeptides Co., Ltd., Shanghai, China) as described previously [25]. An ELISA kit for PGE_2_ was purchased from Cayman Chemical Co. (Ann Arbor, MI, USA). ELISA kits for tumor necrosis factor (TNF)-α, interleukin (IL)-1β, IL-6 and IL-12 measurement were obtained from Neobioscience Technology Company (Guangdong, China). RNAfast200 Trizol reagent was purchased from Fastagen Biotech (Shanghai, China). The cell lysis buffer was obtained from Biocolors (Shanghai, China). A KeyGEN Nuclear and Cytoplasmic Protein Extraction Kit was purchased from KeyGen Biotech (Nanjing, China). The bicinchoninic acid (BCA) reagent was obtained from Auragene Bioscience Corporation, Inc. (Changsha, China). Antibodies against inducible nitric oxide (iNOS), cyclooxygenase-2 (COX-2) and NF-κB/p65, as well as an Alexa Fluor 488-conjugated secondary anti-mouse antibody, were provided by Cell Signaling Technology (Beverly, MA, USA). Antibody against TLR4 was purchased from Abcam Company (Cambridge, UK). Hilyte Fluo™ 488-labled β-amyloid (1-42) (FL-Aβ) was purchased from AnaSpec, Inc. (San Jose, CA, USA) and LysoTracker Red DND-99 was furnished from Molecular Probes (Invitrogen, MO, USA).

### 4.2. Preparation of AdO

AdO was prepared from sodium alginate by a reaction with a 5% H_2_O_2_ solution at 90 °C for 2 h as described in our previous work [23]. The average molecular weight of AdO is about 1500 Da that detected by size exclusion chromatography (SEC) with multi-angle laser light scattering (MALLS).

### 4.3. Cell Culture

The BV2 microglia were cultured in DMEM supplemented with 10% FBS, 100 U/mL penicillin and 100 μg/mL streptomycin at 37 °C in a humidified incubator with 5% CO_2_

### 4.4. Cytotoxicity Assay

The viability of the BV2 cells treated with AdO was evaluated using the CCK-8 assay. Briefly, the cells were pretreated with AdO (100–1000 μg/mL) for 2 h and then incubated with LPS/Aβ for 24 h or treated with AdO (50–1000 μg/mL) for 24 h. The medium was removed and the cells were incubated with 0.5 mg/mL of the CCK-8 solution. After this incubation, the absorption was measured at 540 nm using a microplate reader (Molecular Devices, LLC, Sunnyvale, CA, USA).

### 4.5. Measurement of NO and PGE_2_

The BV2 microglia were pretreated with AdO (50–500 μg/mL) for 2 h and then stimulated with LPS (0.5 μg/mL) for 24 h. The accumulated nitrite in the culture supernatants was measured using the Griess reaction method as described in our previous work [23]. PGE_2_ production was evaluated using an ELISA kit according to the manufacturer’s instructions.

### 4.6. Reverse Transcription Polymerase Chain Reaction (RT-PCR)

RNA was prepared using RNAfast200 Trizol reagent. Total RNA (1 µg) was used for reverse transcription to produce the cDNAs. The iNOS and COX-2 genes were amplified from the cDNA using PCR. The following PCR primers are used in this work: iNOS: Fwd 5′-CAA CCA GTA TTA TGG CTC CT-3′; Reverse 5′-GTG ACA GCC CGG TCT TTC CA-3′. COX-2: Fwd 5′-CCA CTT CAA GGG AGT CTG GA-3′; Reverse 5′-AGT CAT CTG CTA CGG GAG GA-3′. β-Actin: Fwd 5′-GGA GAA GAT CTG GCA CCA CAC C-3′; Reverse 5′-CCT GCT TGC TGA TCC ACA TCT GCT GG-3′.

### 4.7. Western Blot Analysis

The BV2 cells were pretreated with AdO for 2 h and then stimulated with LPS (0.5 μg/mL) for 24 h. The cells were lysed in lysis buffer. To determine the effect of AdO on the nuclear translocation of NF-κB p65, the nuclear and cytoplasmic proteins were extracted by a KeyGEN Nuclear and Cytoplasmic Protein Extraction Kit. The protein concentrations were determined using the bicinchoninic acid (BCA) reagent. For Western blot analysis, 50 μg of proteins was separated by 12.5% SDS-PAGE. Then, the proteins were transferred onto a polyvinylidene difluoride (PVDF) membrane (Amersham Pharmacia Biotech, England, UK) and subsequently blocked in 10% skimmed milk in Tris-buffered saline containing 0.1% Tween 20 (TBST). After the membranes were washed adequately, they were incubated with anti-mouse iNOS (1:1000), anti-mouse COX-2 (1:1000), anti-TLR4 (1:1000), or anti-NF-κB/p65 (1:1000) antibodies in 5% skimmed milk in TBST at 4 °C overnight. The membranes were then washed three times with TBST and incubated with a horseradish peroxidase-conjugated secondary antibody at 37 °C for 2 h. All Western blot assays were performed at least three times.

### 4.8. Measurement of Cytokines

The BV2 microglia were pretreated with AdO (50–500 μg/mL) for 2 h and then treated with LPS (0.5 μg/mL) or Aβ (10 μM) for 24 h. The levels of TNF-α, IL-6, IL-1β and IL-12 were measured using ELISAs according to the manufacturer’s protocols.

### 4.9. Immunofluorescence Analysis

TLR4 expression and nuclear localization of NF-κB/p65 were detected by immunofluorescence analysis. The BV2 cells (4 × 10^5^ cells/well) were cultured on sterile glass coverslips in 6-well culture dishes. After pretreatment with AdO (500 μg/mL) and stimulation with 0.5 μg/mL LPS, the cells were fixed with 4% paraformaldehyde in PBS. Then, the cells were washed with PBS and permeabilized with 0.2% Triton X-100 in PBS. After 60 min of incubation with 1% (w/v) goat serum in PBS, the cells were incubated with anti-TLR4 or anti-NF-κB p65 antibody diluted in PBS (1:200) at 4 °C overnight, washed and then incubated with an Alexa Fluor 488-conjugated secondary anti-mouse antibody for 2 h at 37 °C. The cells were incubated with DAPI (5 μg/mL) for 15 min to reveal the nuclei. The immunofluorescence analysis was carried out by a Fluoview FV1000 laser scanning confocal microscope (Olympus, Tokyo, Japan).

### 4.10. Cell Morphology

The BV2 cells (4 × 10^5^ cells/well) were seeded onto coverslips placed in 35 mm × 35 mm culture dishes. The cells were pretreated with AdO (50–500 μg/mL) and were stimulated with LPS or Aβ for 24 h. Dark-field microscopy was used to examine the cell morphology of BV2 cells using an Olympus BX51 upright optical microscope (Tokyo, Japan). The morphological changes of the cells were monitored using a 40× objective, and images were captured using a DP70 camera (Olympus, Tokyo, Japan). Subsequently, the dark field images were analyzed using ImageJ software (National Institutes of Health, Bethesda, USA).

### 4.11. Phagocytosis Assay

AuNPs were prepared as described in our previous work [43]. Briefly, the cells (4 × 10^5^ cells/well) were treated with AdO (50–500 μg/mL) for 20 h. After three washes, the cells were incubated with a solution of 1 pM AuNPs (100 nm diameter) for the indicated times. The cells were then washed, fixed in 4.0% (w/v) paraformaldehyde and visualized using dark-field microscopy.

In additional studies, the cells (4 × 10^5^ cells/well) were treated with AdO (50 μg/mL) for 20 h and incubated with 500 nM FL-Aβ for 4 h. TLR4 was blocked using anti-TLR4 (10 μg/mL) at 37 °C for 2 h prior to AdO treatment. To reveal the lysosomes and cytoskeleton of the cells, the BV2 cells were fixed and labeled with LysoTracker Red DND-99 (500 nM) and FITC-phalloidin (800 nM) for 1 h. Then, the cells were observed using a Fluoview FV1000 laser scanning confocal microscope (Olympus, Tokyo, Japan). The fluorescence intensity of the cells was analyzed using ImageJ software.

### 4.12. Flow Cytometric Analysis

The BV2 cells were plated in 24-well culture plates (1 × 10^5^ cells/well). The cells were pretreated with AdO (50 μg/mL) for 20 h and then incubated with FL-Aβ for 4 h. TLR4 was blocked by treating with anti-TLR4 (10 μg/mL) at 37 °C for 2 h prior to the AdO treatment. The flow cytometric measurements were carried out using a fluorescence-activated cell sorting (FACS) system 145 (Becton Deckinson, San Jose, CA, USA). Subsequently, the cells were washed adequately and the fluorescence was compared to untreated controls using a total of 10,000 recorded events for each sample.

### 4.13. Statistical Analysis

The data for all experiments are presented as the means ± SD. One-way analysis of variance (ANOVA) and Student’s t-tests were used to determine any significant differences. *p* values < 0.05 were considered to be significant. Each experiment was repeated at least three times.

## 5. Conclusions

Alginate is a natural polysaccharide derived from various kinds of marine brown algae. Previous studies demonstrated that AdO exhibits notably diverse pharmacological activities [15,16,18,19]. However, to the best of our knowledge, this is the first work to explore the effect of AdO on microglia-mediated inflammatory responses and microglial phagocytosis of Aβ. The results of this study revealed dual effects of AdO on BV2 microglial cells. First, AdO exerted an inhibitory effect on the LPS/Aβ-activated inflammatory response, and second, AdO promoted the microglial phagocytosis of Aβ. Therefore, the current work proposed that AdO is a potentially therapeutic nutraceutical for treating AD or other neurodegenerative disease.

## Supplementary Files

Supplementary File 1
