# Supplementary material for: Alginate-Derived Oligosaccharide Inhibits Neuroinflammation and Promotes Microglial Phagocytosis of β-Amyloid
Source: Mar Drugs. 2015 Sep 16;13(9):5828–46. doi: 10.3390/md13095828 (PMC4584357; doi:10.3390/md13095828)
Supplement: Supplementary File 1 [file marinedrugs-13-05828-s001.doc]

Supplementary Information


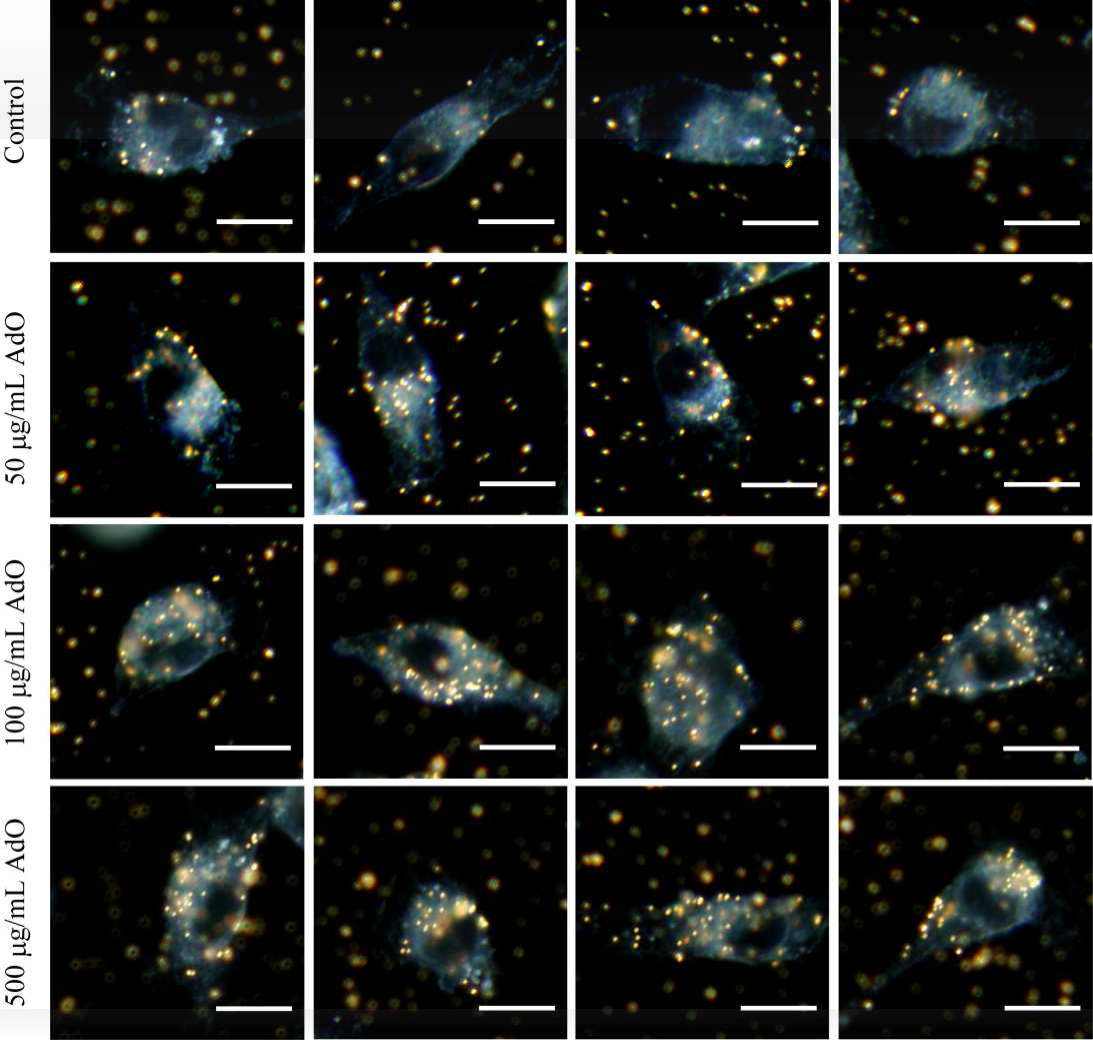


**Figure S1.** AdO promoted the phagocytosis of the AuNPs in single BV2 cells. The cells with accumulated AuNPs were examined using dark-field microscopy (40×), scale bar = 20 μm. Each row shows four cells randomly selected from the samesample. Row 1: AuNPs in untreated BV2 cells; Row 2: AuNPs in BV2 cells treatedwith 50 mg/mL AdO; Row 3: AuNPs in BV2 cells treated with 100 mg/mL AdO; Row4: AuNPs in BV2 cells treated with 500 mg/mL AdO. The images shown here werefrom three independent experiments.


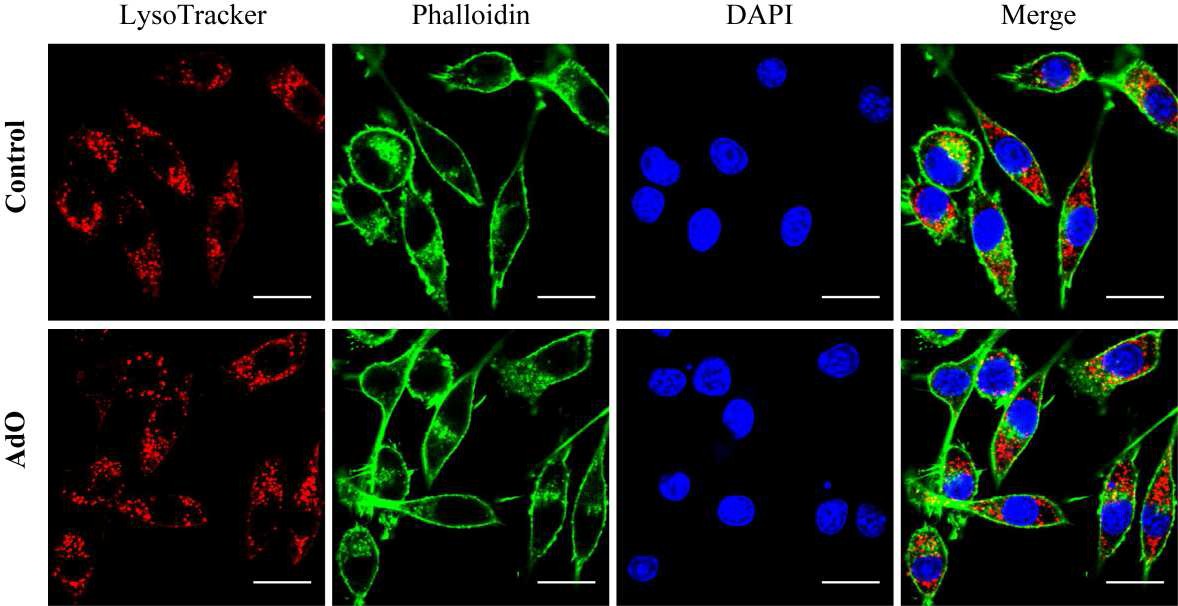


**Figure S2.** The effect of AdO on the lysosomes and actin cytoskeleton in BV2 cells. The cells were treated with AdO for 20 h. The lysosomes and cytoskeleton werelabeled with LysoTracker Red DND-99 and FITC-phalloidin, respectively. The cellnuclei were stained by DAPI. The images were acquired using laser scanningconfocal microscopy (60×), scale bar = 20 μm. The images shown were from threeindependent experiments.

© 2015 by the authors; licensee MDPI, Basel, Switzerland. This article is an open access article distributed under the terms and conditions of the Creative Commons Attribution license (http://creativecommons.org/licenses/by/4.0/).
